# Isolation of 3*β*‐Hydroxylupenal From *Ceriops decandra* Leaves and Evaluation of In Vivo Analgesic and Anti‐Inflammatory Effects Supported by Molecular Docking

**DOI:** 10.1155/bmri/4730586

**Published:** 2026-05-18

**Authors:** Asef Raj, Sania Ashrafi, Md. Sakhawat Hossain, Md. Saiful Islam, Muhammad Abdullah Al-Mansur, Abu Bin Ihsan, Md. Ruhul Kuddus, Firoj Ahmed

**Affiliations:** ^1^ Department of Pharmaceutical Chemistry, University of Dhaka, Dhaka, Bangladesh, du.ac.bd; ^2^ School of Pharmacy, BRAC University, Dhaka, Bangladesh, bracu.ac.bd; ^3^ Pharmaceutical Sciences Research Division, Bangladesh Council of Scientific and Industrial Research (BCSIR), Dhaka, Bangladesh, bcsir.gov.bd; ^4^ Institute of National Analytical Research and Service, Bangladesh Council of Scientific and Industrial Research (BCSIR), Dhaka, Bangladesh, bcsir.gov.bd; ^5^ Department of Pharmacy, Eastern University, Dhaka, Bangladesh, eastern.edu

**Keywords:** Bangladesh, ethnomedicine, inflammation, lupeol, mangrove, pain, phytochemistry, triterpenoids

## Abstract

*Ceriops decandra* (Griff.) Ding Hou, locally known as “Goran” in Bangladesh, is a recognized mangrove plant of the Sundarbans. Coastal communities living in tidal areas traditionally use this plant for treating various ailments. While numerous biological activities of this plant have been reported, its leaves of Bangladeshi origin have not been phytochemically characterized in detail. Phytochemical analysis led to the isolation and identification of five compounds, namely lupeol (**1**), 3*β*‐*E*‐coumaroyllupeol (**2**), betulin (**3**), 3*β*‐hydroxylupenal (**4**), and *β*‐sitosterol (**5**). ^1^H and ^13^C NMR spectroscopic analyses and comparison of spectral data with published literature were performed to identify these compounds. Compound **4** is reported here for the first time from *C. decandra*, whereas Compounds **1–3** are reported for the first time from the leaves of Bangladeshi‐origin *C. decandra*. The in vivo assays were undertaken to support the plant′s previously recognized analgesic and anti‐inflammatory relevance and to explore whether the newly characterized constituents may contribute to these effects. The crude methanolic leaf extract of *C. decandra* at the dose of 400 mg/kg body weight showed potent central and peripheral analgesic effects in acetic acid–induced writhing, formalin‐induced licking, hot plate, tail immersion, and tail clip test in mice. The extract at 200 mg/kg also produced significant anti‐inflammatory activity in the carrageenan‐induced paw edema model, with peak inhibition of 29.81% at 3 h postcarrageenan. Molecular docking with mu, COX‐1, and COX‐2 receptors revealed that Compounds **2** and **4** may have stronger analgesic potential than diclofenac sodium. The isolated compounds showed favorable absorption, distribution, metabolism, excretion, and toxicity (ADMET) properties for drug development. In addition, Prediction of Activity Spectra for Substances (PASS) analysis indicated high predicted anticancer potential for the isolated compounds, warranting future investigation.

## 1. Introduction

The use of plants for medicinal purposes is deeply rooted in human history. Throughout history, plants have been used to treat a variety of ailments and diseases. In modern times, the use of medicinal plants has become increasingly popular due to their natural and affordable properties [1]. The active components of many commonly used traditional medicines like strychnine, emetine, piperine, caffeine, quinine, colchicine, coniine, etc. are the contribution of phytochemical research [2].

*Ceriops decandra* (Griff.) Ding Hou (Family: Rhizophoraceae, Bengali name: Goran) is a small to medium‐sized (up to 35 m), evergreen, straight, and columnar tree under the Rhizophoraceae family that can reach its maximum height under favorable conditions. The plant is commonly found along the eastern coastlines of Bangladesh, India, southwestern Thailand, and the western part of the Malay Peninsula. This plant can be found in abundance in the Sundarbans of Bangladesh. However, the plant′s distribution is becoming more constrained and scarce as a result of the accelerating rate of deforestation. According to the International Union for Conservation of Nature (IUCN) Red List, *C. decandra* is currently classified as “near threatened” [3].

Previous studies on *C. decandra* have identified mainly terpenoid‐rich constituents and reported several biological activities, suggesting that its secondary metabolites may contribute to its pharmacological effects [4–9]. *C. decandra* is known for its strong therapeutic effects, many of which have been verified via multiple in vitro and in vivo tests [3, 5]. Studies have shown that various components of *C. decandra* possess antiplasmodial, anti‐HIV, antimicrobial, antidiabetic, antioxidant, anti‐inflammatory, antidiarrheal, and anticoagulant activities [10–13]. The various parts of the plant are traditionally used by the local health practitioners in tidal coastal communities as remedies for a wide range of ailments. According to available ethnomedicinal literature, the plant is primarily used to treat viral infection, amoebiasis, hepatitis, ulcer, diarrhea, vomiting, hemorrhage, pain, wounds, boils, angina, diabetes, dermatological conditions, pain, and inflammation [9, 10, 14–17]. However, these earlier reports mainly establish the therapeutic relevance of *C. decandra* at the extract or ethnomedicinal level and do not identify the responsible phytoconstituents nor do they provide a phytochemical profile of Bangladeshi‐origin leaves linked to pain‐ and inflammation‐related activity.

*C. decandra* was selected for the present investigation for three related reasons. First, the species has documented ethnomedicinal relevance in coastal communities, particularly for pain‐ and inflammation‐related conditions. Second, prior studies on *C. decandra* leaf material have already indicated antinociceptive, anti‐inflammatory, and related pharmacological potential, suggesting that the leaves are a biologically relevant organ for further study. Third, despite this therapeutic background, the phytochemical profile of Bangladeshi‐origin leaves has remained insufficiently characterized at the constituent level. Although the anti‐inflammatory and anti‐ulcer activity of the methanolic leaf extract and isolated lupeol from *C. decandra* leaves have been reported previously, the broader phytochemical composition of Bangladeshi‐origin leaves remained unresolved, and the analgesic profile of the extract had not been examined across multiple complementary nociceptive models.

In this context, this study aimed to address a specific phytochemical knowledge gap. The leaves of Bangladeshi‐origin *C. decandra* were investigated phytochemically, leading to the first report of 3*β*‐hydroxylupenal from this species and the first isolation of three triterpenoids from its Bangladeshi leaves. The crude extract was then evaluated in vivo for analgesic and anti‐inflammatory effects to examine its previously reported analgesic and anti‐inflammatory activity, whereas molecular docking, ADMET (absorption, distribution, metabolism, excretion, and toxicity), and PASS ((Prediction of Activity Spectra for Substances) analyses were used to examine whether the newly characterized constituents could plausibly contribute to the observed bioactivity. We hypothesized that bioactive constituents isolated from Bangladeshi‐origin *C. decandra* leaves contribute to its analgesic and anti‐inflammatory effects and would therefore be expected to manifest as significant in vivo activity of the crude extract and favorable docking interactions of the isolated compounds with *μ*‐opioid, COX‐1, and COX‐2 targets. Given the conservation sensitivity of this mangrove species and the broader degradation pressures affecting Sundarbans mangroves, the present work focused on leaf material rather than destructive sampling of bark, stem, or roots, and any future scale‐up should rely on regulated, nondestructive collection and, preferably, cultivated or restoration‐linked biomass sources.

## 2. Method

### 2.1. Collection and Identification of Plant Material

*C. decandra* leaves were obtained from Bangladesh′s Sundarbans mangrove area (Notabaki, Sundarban, Satkhira, Bangladesh) in October 2022. A taxonomist from the Jahangirnagar University Herbarium verified the plant sample′s authenticity and assigned it the Accession Number JUH‐10121. For future reference, a specimen of the plant was stored at the herbarium.

### 2.2. Crude Extract Preparation

After collection, the leaves were separated from the stalks. They were cleaned properly and then shade‐dried for 2 weeks. A high‐capacity grinder was used to crush the dry leaves into coarse powder. The grinding process yielded 1.5 kg of coarse powder, which was subsequently placed in a clean round‐bottom flask. The powder was then immersed in approximately 4 L of distilled methanol for a period of 15 days, during which it was periodically agitated and stirred. Following that, the mixture was filtered through new cotton plugs and Whatman filter paper Number 1. To obtain a semisolid crude extract, the filtrate′s volume was reduced using an EYELA rotary evaporator under low temperature (not exceeding 40°C) and pressure conditions. After completing several extraction cycles, the resulting dried crude extract yielded a total mass of 109.4 g (yield 7.3%).

### 2.3. Fractionation of the Crude Extract

Then, using the modified Kupchan partitioning method, 5 g of crude extract was divided into n‐hexane, ethyl acetate, and water soluble fractions [1]. The fractionation process was repeated multiple times, and a total of 65 g of crude methanolic extract was partitioned to yield approximately 10 g of n‐hexane fraction, 19 g of ethyl acetate fraction, and 32 g of water‐soluble fraction. All in vivo pharmacological experiments were performed using the crude methanolic extract of *C. decandra* leaves (CDME), whereas the solvent‐partitioned fractions were used only for phytochemical isolation.

### 2.4. Isolation of Chemical Compounds

An aliquot of the n‐hexane soluble partitionate (6 g) was fractionated using column chromatography on column grade silica powder (Kieselgel 60, particle size 0.06–0.2 mm, MW 60.09 g/mol). Elution began with 100% n‐hexane, and the polarity of the mobile phase was gradually increased by adding ethyl acetate and methanol. The eluates were collected in beakers and screened using TLC. In total, 58 portions (100 mL each) were collected. Fractions that displayed similar TLC behavior were mixed, and 23 subfractions were obtained. Compound **1** was isolated from the Subfraction 2L by crystal wash with n‐hexane. Compounds **2** and **5** were collected by preparative TLC from the Subfractions 2A and 2N, respectively.

The ethyl acetate soluble partitionate was then fractionated via vacuum liquid chromatography (VLC) with VLC grade Kieselgel 60H silica gel. The column was initially eluted with n‐hexane, and the solvent′s polarity was gradually enhanced by adding more polar solvents like chloroform, ethyl acetate, and methanol. The eluates were collected in beakers and screened using TLC. In total, 27 portions (100 mL each) were collected. Fractions that displayed similar TLC behavior were mixed, and eight subfractions were obtained. Compounds **3** and **4** were collected by preparative TLC from the Subfractions 12G and 12D, respectively.

### 2.5. Characterization of the Isolated Compounds

Proton and carbon nuclear magnetic resonance measurements (^1^H and ^13^C NMR) were carried out using Bruker 400 and 600‐MHz equipment for the characterization of the isolated compounds. Deuterated chloroform, CDCl_3_, was used as a solvent to obtain the spectra. Coupling constants were measured in Hertz (Hz), whereas parts per million (ppm) was used to express chemical shifts.

### 2.6. In Vivo Study

#### 2.6.1. Test Animal Model

Swiss albino mice of both sexes, weighing between 25 and 30 g and aged 4–5 weeks, were obtained from the animal facility at the International Centre for Diarrheal Disease Research, Bangladesh (icddr,b). Mice were housed in polypropylene cages with uniform environmental conditions, including a 12‐h light–dark cycle, 60%–70% relative humidity, and a regulated temperature of 24C ± 2^°^C. They had unrestricted access to icddr,b formulated rodent diet and water. The animals were allowed to adjust to the laboratory environment for a week before the experiments began. After acclimatization, mice were randomly assigned to the experimental groups (*n* = 6 per group). Male and female mice were distributed across the experimental groups in an approximately equal manner, and allocation was randomized to minimize sex‐related allocation bias. The study was not powered for a separate sex‐stratified analysis, and outcomes were therefore analyzed collectively. The sample size (*n* = 6 per group) was selected in line with commonly used group sizes in comparable rodent analgesic and anti‐inflammatory studies, while also reflecting the principle of reduction, which aims to use the smallest number of animals necessary to achieve the scientific objective [18, 19]. Behavioral scoring and paw‐volume measurements were performed by an investigator blinded to group allocation to minimize observer bias. All animal experiments were approved by the Ethical Review Committee of the Faculty of Pharmacy, University of Dhaka (Ref. No. Fa. Ph. E/057/2025; Approval Date: May 13, 2025) and were conducted in accordance with the principles of the Guide for the Care and Use of Laboratory Animals.

#### 2.6.2. Acute Oral Toxicity Study

An acute oral toxicity study of the crude methanolic extract of *C. decandra* leaves was carried out in mice according to the OECD Guideline 423 (Acute Toxic Class Method) [20]. Healthy Swiss albino mice were fasted overnight before dosing, with free access to water. The extract was suspended in 1% Tween‐80 in distilled water and administered orally by gavage at a limit dose of 2000 mg/kg body weight. After administration, the animals were observed individually during the first 30 min, periodically during the first 4 h, and then once daily for 14 days for mortality and clinical signs of toxicity, including changes in skin and fur, eyes, mucous membranes, salivation, tremors, convulsions, lethargy, sleep, diarrhea, posture, locomotor activity, and feeding behavior. Body weight was recorded on Days 0, 7, and 14. At the end of the observation period, the extract was considered tolerable at the tested dose level in the absence of mortality or evident toxic manifestations. These findings were used to support the selection of pharmacological test doses for the subsequent in vivo experiments.

#### 2.6.3. Dose Selection

The extract doses were selected as graded pharmacological test levels based on the results of the acute oral toxicity study and on dose ranges commonly used for crude plant extracts in rodent analgesic and anti‐inflammatory bioassays [21–24]. In the present study, the crude methanolic extract was found to be tolerable at an oral limit dose of 2000 mg/kg in the acute oral toxicity study, and the pharmacological doses were therefore selected well below that level. Accordingly, 200 and 400 mg/kg were employed in the analgesic models to assess dose responsiveness, whereas lower oral doses (100 and 200 mg/kg) were used in the carrageenan‐induced paw edema model as a conservative screening range for anti‐inflammatory activity. The crude methanolic extract was administered intraperitoneally (i.p.) in the analgesic assays to achieve more rapid and consistent systemic exposure during acute nociceptive testing and to minimize variability associated with gastrointestinal absorption after oral dosing.

#### 2.6.4. Analgesic Activity

##### 2.6.4.1. Acetic Acid–Induced Writhing Test

Writhing test was carried out following the protocol outlined by Mondal et al. [25]. The negative control group received 1% Tween‐80 (10 mL/kg body weight, orally) in distilled water, whereas the positive control group received diclofenac sodium (10 mg/kg body weight). The treatment groups were administered 200 and 400 mg/kg body weight of CDME. All treatments were given i.p. 30 min before injecting 0.6% acetic acid (10 mL/kg body weight) into each mouse. Writhing movements (abdominal contractions) were observed and recorded for a duration of 15 min, commencing 5 min after the administration of acetic acid. The analgesic effects of the test compounds were indicated by the reduced writhing response observed in the treated groups compared to the control group. The formula below was used to compute the antinociceptive effect:

Inhibition %=Average writhing of control−Average writhing of test sampleAverage writhing of control×100.

##### 2.6.4.2. Formalin‐Induced Licking Test

Paw‐licking assay in mice was performed according to the methods described by Santos et al. and Lucarini et al. [26, 27]. The negative control group received 1% Tween‐80 (10 mL/kg body weight, orally) in distilled water, whereas the positive control group received diclofenac sodium (10 mg/kg body weight). CDME was administered i.p. to the test groups at doses of 200 and 400 mg/kg body weight. After 30 min, a 2.5% formalin solution (20 *μ*L) was injected into the subplantar area of the left hind paw. The mice were placed in translucent boxes, and the amount of time the mice spent licking and biting the injected paw was noted as a pain indication. Observations were made during the early phase (0–5 min, neurogenic phase) and the late phase (15–30 min, inflammatory phase) after formalin injection. The test samples exhibited analgesic effects if the licking behavior in the treated animals was reduced compared to the untreated control group. The following formula was used to determine the pain inhibition ratio:

Inhibition %=Average licking of control−Average licking of sampleAverage licking of control×100.

##### 2.6.4.3. Hot‐Plate Test

The hot‐plate test in mice was conducted using the procedure described by Lucarini et al. [27]. The control group received 1% Tween‐80 (10 mL/kg body weight, orally) in distilled water, whereas the positive control group was treated with diclofenac sodium (10 mg/kg body weight). The treatment groups were administered CDME at doses of 200 and 400 mg/kg body weight via i.p. injection. After 30 min, the mice were subjected to a thermal pain stimulus by placing them on a hot plate kept at 55C ± 0.5^°^C. Paw licking or jumping off the plate was noted as an example of pain response behaviors. Baseline reaction times (Tb) were measured prior to treatment by recording paw‐licking or jumping responses at 0 and 10 min, with the average serving as the initial reaction time. Following a 30‐min latency interval, posttreatment response times (Ta) were assessed at 0, 30, 60, 90, and 120 min after the extract or medication was administered. The analgesic effects of the extract and the standard drug were compared with the control group by evaluating changes in reaction times. The percentage change in reaction time (analgesic activity) was calculated for each animal using its own pretreatment baseline latency (Tb) and posttreatment latency (Ta), as indicated in the formula

Percentage analgesic activity=Ta−Tb/Tb×100%.

##### 2.6.4.4. Tail Immersion

The tail immersion test in mice was conducted following the protocols described by Asongalem et al. and Lucarini et al. [27, 28]. Diclofenac sodium (10 mg/kg body weight) was administered to the positive control group, whereas 1% Tween‐80 (10 mL/kg body weight, orally) in distilled water was given to the control group. The test groups were given the CDME at doses of 200 and 400 mg/kg body weight via i.p. injection. After a 30‐min interval, the distal 3 cm of each mouse′s tail was immersed in a water bath set at 55C ± 0.5^°^C, and the time it took for the mouse to withdraw its tail was recorded as the pain response using a stopwatch. To determine baseline reaction times (Tb), two measurements were taken for each mouse at 0 and 10 min. The average of these readings served as the baseline. The reaction times (Ta) for the treatment groups were recorded at 0, 30, 60, 90, and 120 min after a 30‐min waiting period following the administration of the extract and drug. The following formula was used to evaluate analgesic activity:

%analgesic activity=Ta−Tb/Tb×100%.

##### 2.6.4.5. Tail Clip Method

The tail clip test in mice was performed based on the method outlined by Sekhar et al. [29]. Initially, each mouse was examined by connecting a metal artery clip to the base of its tail, with the clip′s jaws cushioned by thin rubber tubes. The pressure was adjusted to ensure that all control mice reacted to the stimulus. Mice were removed from the trial if they did not attempt to remove the clip within 10 s. Five groups, each including six mice, were created from the selected mice. The control group was given 1% Tween‐80 (10 mL/kg body weight, orally) in distilled water, the positive control group was given diclofenac sodium (10 mg/kg body weight), and the test groups were given the CDME at doses of 200 and 400 mg/kg body weight via i.p. injection. Analgesic activity was assessed 1.5 h postadministration by applying the tail clip. A positive analgesic effect was recorded provided that the mouse did not try to remove the clip within 10 s in four consecutive trials spaced 2 min apart. The mean analgesic effect was calculated using the following formula:

%analgesic activity=Mean response latency of test group−Mean response latency of control groupMean response latency of control group×100.

#### 2.6.5. In Vivo Anti‐Inflammatory Study

The carrageenan‐induced hind paw edema model was employed to test acute inflammation in mice, according to the procedure described by [30]. The carrageenan‐induced paw edema model, although classically established in rats, has also been validated in mice and remains suitable for acute anti‐inflammatory screening in murine studies [31, 32]. Mice were used here to maintain species consistency across the in vivo pharmacological assays, allowing a more integrated comparison of analgesic and anti‐inflammatory responses within the same experimental framework, while also reducing the need to introduce an additional animal species. In the positive control group, indomethacin (10 mg/kg body weight) was given orally, whereas the control group received 1% Tween‐80 in normal saline (10 mL/kg). CDME was given to the experimental groups at levels of 100 and 200 mg/kg body weight. Acute inflammation was caused by a subplantar injection of 0.1‐mL carrageenan suspension (in 1% Tween‐80 normal saline) into the right hind paw 1 h after the test drugs were administered orally. A plethysmometer was used to measure paw volume at various time points (before injection, as well as 1, 2, 3, and 4 h after). The percentage inhibition of inflammation generated by the extract was calculated using the following equation:

%suppresion of inflammation=Vc−Vt/Vc×100.

In this formula, Vc represents the average level of inflammation observed in the control group, whereas Vt represents the average level of inflammation in the test group.

### 2.7. Molecular Docking Study

#### 2.7.1. Ligand Preparation

The three‐dimensional molecular structures of lupeol, betulin, and celecoxib were obtained from the PubChem database (https://pubchem.ncbi.nlm.nih.gov/) in SDF file format (PubChem CID: 259846, 72326, and 2662, respectively). Since 3*β*‐*E*‐coumaroyllupeol **(2)** and 3*β*‐hydroxylupenal **(4)** are not enlisted in the PubChem database, their 2D chemical structures were drawn in the ChemDraw Professional (Version: 16.0.1.4), and the files were saved in SDF format [33].

#### 2.7.2. Protein Preparation

Considering the traditional use of *C. decandra* leaves for treating pain and inflammation [3], the human mu‐opioid receptor (PDB ID: 5C1M) was chosen to investigate central analgesic activity, whereas COX‐1 (PDB: 6Y3C) and COX‐2 (PDB: 5F1A) were selected as targets to explore peripheral analgesic activity during the docking studies of the isolated compounds. The 3D structures were obtained from the RCSB Protein Data Bank (https://www.rcsb.org). Ligands, water molecules, and heteroatoms were removed using Discovery Studio 2020, nonpolar hydrogen atoms were introduced, and, finally, energy was minimized using Swiss‐PdbViewer [34, 35].

#### 2.7.3. Ligand–Protein Docking

Docking was conducted to get a range of potential conformations and orientations of the ligands at the binding site of the receptor, keeping the receptor rigid. Receptors′ interactions and binding patterns with phytochemicals were analyzed using Vina Wizard of PyRx (Version 0.8). The initial step involved loading and formatting the protein to correspond with the target macromolecule. Subsequently, specific amino acids were selected based on their established IDs in the literature to ensure ligand binding that was specific to the target.

For site‐specific docking with the mu‐opioid receptor, the amino acids Asp147, Tyr148, Met151, Lys233, Trp293, Ile296, His297, Val300, Ile322, Gly325, and Tyr326 from the A chain were chosen as key target residues [36]. For the COX‐1 receptor, Arg120, Phe205, Phe209, Tyr355, Ile523, Ser530, Leu534, and Phe381 from A chain [37] and for the COX‐2 receptor, His90, Arg120, Gln192, Val349, Leu352, Ser353, Tyr355, Leu359, Tyr385, Trp387, Arg513, Ala516, Phe518, Val523, Gly526, Ala527, and Leu531 of A chain of were selected for site‐targeted docking [38]. Diclofenac sodium, ibuprofen, and celecoxib were used as standards to assess their affinities with these receptors.

Next, the PDB files of the ligands were imported and converted to pdbqt format. Finally, the grid box coordinates were centered on the reported active‐site cavity and sized to encompass all selected binding‐site residues surrounding the cocrystallized ligand‐binding region. For the mu‐opioid receptor, the grid box was set with coordinates X = −0.9837, Y = 14.1814, and Z = −57.4453 and dimensions X = 19.4747 Å, Y = 18.8939 Å, and Z = 20.8933 Å. For the COX‐1 receptor, the grid box was defined at X = −35.1684, Y = −46.0841, and Z = 1.5145, with dimensions X = 23.6768 Å, Y = 28.6475 Å, and Z = 21.4102 Å. Lastly, the grid box for the COX‐2 receptor was centered at X = 44.2965, Y = 21.5740, and Z = 242.2764, with dimensions X = 26.0091 Å, Y = 22.5350 Å, and Z = 27.5048 Å.

The exhaustiveness was kept unchanged at 8. The remaining parameters were kept unchanged. To validate the docking protocol, the native cocrystallized ligand was removed and redocked into the same binding pocket using the same docking parameters. Reproduction of the crystallographic binding pose with an acceptable RMSD (< 2 Å) supported the suitability of the selected grid box and docking settings for the present analysis [39]. After the docking was complete, the protein–ligand interactions were visualized using 2D and 3D configurations in BIOVIA Discovery Studio Visualizer (Version 21.1.0.20298).

#### 2.7.4. ADMET Prediction

In this study, the Swiss ADME online server (http://www.swissadme.ch/) was used to predict the absorption, distribution, metabolism, and excretion properties of the test compounds, whereas the ProTox‐II online server (https://tox-new.charite.de/protox _II/) was used to predict their toxicities. The canonical SMILES of lupeol and betulin were copied from PubChem database (https://pubchem.ncbi.nlm.nih.gov/) and pasted on the servers for prediction. The chemical structures of 3*β*‐*E*‐coumaroyllupeol **(2)** and 3*β*‐hydroxylupenal **(4)** were drawn on the servers for prediction as they are not enlisted in PubChem [40].

#### 2.7.5. PASS Study

The PASS computer program was selected to predict analgesic and other possible pharmacological activities of the compounds being tested. For this research, the PASS online platform (http://www.way2drug.com/passonline/) was employed. The canonical SMILES of the studied compounds were pasted in the server for prediction [41].

### 2.8. Statistical Analysis

The statistical data were reported as mean ± standard error of mean (SEM). IBM SPSS Statistics Version 27 was used for data analysis. Data were assessed for approximate normality using the Shapiro–Wilk test and for homogeneity of variance using Levene′s test prior to multiple‐group comparison. Statistical analysis was performed using one‐way ANOVA followed by Dunnett′s multiple comparison test. Comparisons were made with the control group, with significance levels defined at *p* < 0.05, *p* < 0.01, and *p* < 0.001 [42].

## 3. Results

**Figure 1:**
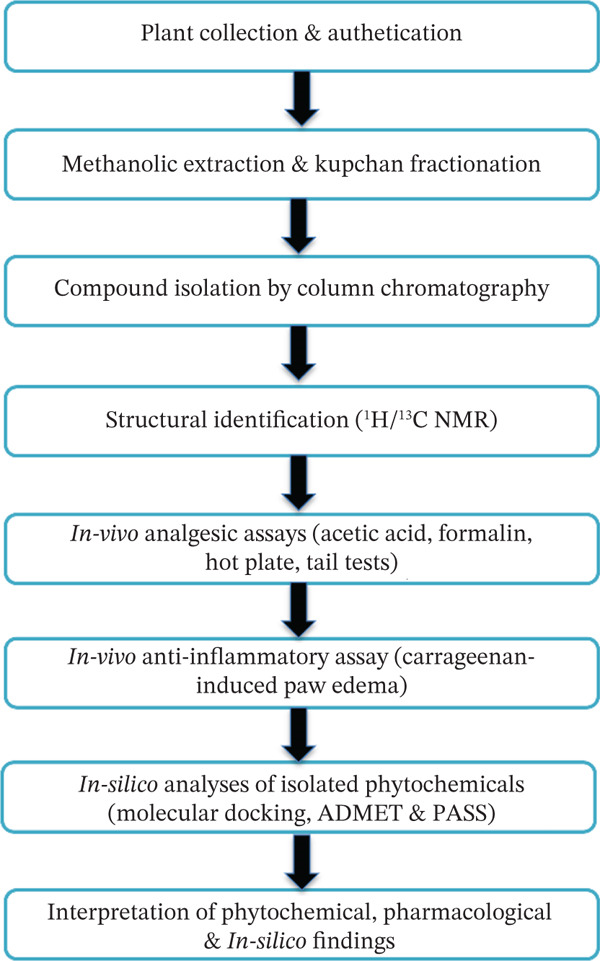
Schematic overview of the experimental workflow used in the present study, from plant collection and phytochemical isolation to in vivo pharmacological evaluation and in silico analysis.

To provide a clear overview of the study design, the experimental workflow involved (Figure 1) collection and authentication of *C. decandra* leaves; methanolic extraction and fractionation; isolation and structural identification of major constituents by chromatographic and NMR analyses; evaluation of the crude methanolic extract in in vivo analgesic and anti‐inflammatory models; and in silico assessment of the isolated compounds through molecular docking, ADMET prediction, and PASS analysis. The results are presented in this same order to facilitate interpretation of the relationship between phytochemical findings and biological activity.

### 3.1. Isolation and Identification of Phytochemicals From *C. decandra* Leaves

Using various chromatographic methods, five distinct compounds were extracted from *C. decandra* leaves. The compounds were identified as lupeol (**1**), 3*β*‐*E*‐coumaroyllupeol (**2**), betulin (**3**), 3*β*‐hydroxylupenal (**4**), and *β*‐sitosterol (**5**) by analyzing their high‐quality ^1^H NMR and ^13^C NMR spectroscopic data and comparing with the published values. The chemical shifts of the compounds in ^1^H NMR spectroscopic data are presented in Tables 1 and 2.

**Table 1:** ^1^H NMR data of Compounds **1–4** in parts per million (400 MHz, CDCl_3_).

Position	Compound 1 (lupeol) *δ*_*H*_(ppm)	Compound 2 (3*β*‐*E*‐coumaroyllupeol) *δ*_*H*_(ppm)	Compound 3 (betulin) *δ*_*H*_(ppm)	Compound 4 (3*β*‐hydroxylupenal) *δ*_*H*_(ppm)
1	0.90 (t), 1.67 (d)	1.00 (m), 1.66 (m)	0.90 (m), 1.65 (m)	0.87 (m), 1.65 (m)
2	1.55 (m), 1.60 (d)	1.59 (m), 1.68 (m)	1.59 (m)	1.56 (m), 1.61 (m)
3	3.18 (dd, *J* = 11.2, 5.2)	4.57 (m)	3.18 (dd, *J* = 11.2, 5.2)	3.18 (dd, *J* = 11.2, 5.2)
5	0.68 (d)	0.82 (m)	0.68 (m)	0.67 (m)
6	1.39 (q), 1.55 (d)	0.74 (m), 1.38 (m)	1.39 (m), 1.53 (m)	1.40 (m), 1.53 (m)
7	1.41 (m)	1.38 (m), 1.43 (m),	1.40 (m)	1.38 (m)
9	1.28 (d)	1.26 (m)	1.27 (m)	1.21 (m)
11	1.24 (q), 1.42 (d)	1.29 (m), 1.40 (m)	1.20 (m), 1.41 (m)	1.21 (m), 1.37 (m)
12	1.06 (q), 1.67 (d)	1.07 (m), 1.65 (m)	1.04 (m), 1.63 (m)	0.91 (m), 1.08 (m)
13	1.67 (t)	1.36 (m)	1.65 (m)	1.66 (m)
15	1.00 (d), 1.71 (t)	0.85 (m), 0.90 (m)	1.05 (m), 1.71 (m)	1.03 (m), 1.70 (m)
16	1.38 (t), 1.49 (d)	1.33 (m), 1.40 (m)	1.20 (m), 1.94 (m)	1.46 (m), 1.52 (m)
18	1.38 (t)	1.38 (m)	1.58 (m)	1.64 (m)
19	2.39 (m)	2.37 (m)	2.38 (m)	2.75 (m)
21	1.33 (m), 1.92 (m)	1.22 (m), 1.31 (m)	1.40 (m), 1.94 (m)	1.24 (m), 2.16 (m)
22	1.20 (m), 1.41 (m)	1.16 (m), 1.36 (m)	1.03 (m), 1.86 (m)	1.39 (m), 1.44 (m)
23	0.97 (s)	0.87 (s)	0.97 (s)	0.96 (s)
24	0.76 (s)	1.03 (s)	0.76 (s)	0.76 (s)
25	0.83 (s)	0.87 (s)	0.83 (s)	0.81 (s)
26	1.03 (s)	0.89 (s)	1.03 (s)	1.02 (s)
27	0.96 (s)	0.94 (s)	0.98 (s)	0.93 (s)
28	0.78 (s)	0.78 (s)	3.32 (d, *J* = 6.8), 3.78 (d, *J* = 7.2)	0.82 (s)
29	4.56 (br. s), 4.68 (br. s)	4.56 (br. s), 4.68 (br. s)	4.56 (br. s), 4.68 (br. s)	5.90 (s), 6.27 (s)
30	1.68 (s)	1.67 (s)	1.68 (s)	9.51 (s)
2 ^′^		6.29 (d, *J* = 16.2)		
3 ^′^		7.57 (d, *J* = 16.2)		
5 ^′^, 9 ^′^		7.42 (d, *J* = 8.7)		
6 ^′^, 8 ^′^		6.82 (d, *J* = 8.7)		

*Note:* Coupling constant, *J*, is reported in hertz only for clearly resolved signals.

**Table 2:** ^1^H NMR data of Compound **5** in parts per million (400 MHz, CDCl_3_).

Position ^a^	Compound 5 (*β*‐sitosterol) *δ*_*H*_(ppm)
3	3.52 (1H, m)
6	5.35 (1H, d, *J* = 6.4 Hz)
18	1.00 (3H, s)
19	0.67 (3H, s)
21	0.92 (3H, d, *J* = 6.5 Hz)
23	5.19 (1H, m)
26	0.82 (3H, d, *J* = 6.4 Hz)
27	0.80 (3H, d, *J* = 6.4 Hz)
29	0.84 (3H, t, *J* = 7.2 Hz)

^a^Only diagnostic ^1^H NMR signals of *β*‐sitosterol are shown.

**Figure 2:**
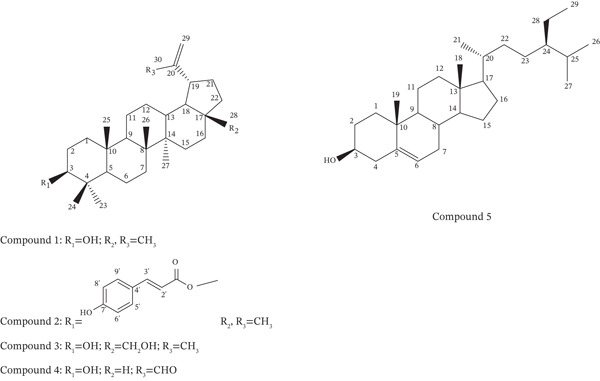
Chemical structure of Compounds **1–5**.

Analysis of the Compound **1** using ^1^H NMR at 400 MHz in CDCl_3_ revealed the presence of seven singlet methyl protons at *δ* 0.76, 0.78, 0.83, 0.96, 0.97, 1.03, and 1.68. Besides, two doublets at *δ* 4.56 (1H, d, *J* = 0.8 Hz) and *δ* 4.68 (1H, d, *J* = 2.4 Hz) indicated the presence of an olefinic moiety (Figure S1). The olefinic group along with seven methyl singlets suggested the presence of a pentacyclic triterpenoid structure. The presence of a one proton double doublet signal at *δ* 3.18 and a typical lupane H_*β*_‐19 proton signal at *δ* 2.39 strongly suggested Compound **1** to be lupeol (Figure 2). Additional verification of the structure was obtained by comparing its spectral information with previously reported values [43].

The ^1^H NMR spectral data of Compound **2** were similar to that of Compound **1** with some additional signals (Figure S2). Two doublets at *δ* 7.42, (2H, d, *J* = 8.7, H‐5 ^′^, H‐9 ^′^) and *δ* 6.82 (2H, d, *J* = 8.7, H‐6 ^′^, H‐8 ^′^) indicated the presence of 1,4‐disubstituted aromatic ring system. The one proton doublets at *δ* 7.57 (1H, d, *J* = 16.2) and *δ* 6.29 (1H, d, *J* = 16.2) indicated the existence of a pair of *trans* olefinic protons (H‐3 ^′^ and H‐2 ^′^, respectively). These signals suggested the presence of a *trans*‐coumaroyl substituent.

Moreover, the oxymethine proton (H‐3) signal was seen to shift to the more downfield region (at *δ* 4.57) compared to that of Compound **1** (*δ* 3.18), which indicated the presence of *trans*‐coumaroyl substituent at C‐3 position. These spectral features suggested the compound to be 3*β*‐*E*‐coumaroyllupeol (Figure 2). Comparison of the compound′s ^1^H NMR spectral data with previously published information for 3*β*‐*E*‐coumaroyllupeol provided additional confirmation of its structural composition [44].

The ^1^H NMR spectral data of Compound **3** were also very similar to the spectrum of Compound 1 (Figure S3). The difference included the presence of six singlet methyl protons signals instead of seven. The signal of H‐28 methyl protons at *δ* 1.68 of Compound **1** was absent in this compound. Moreover, there was the presence of the AB system of oxymethylene protons at *δ* 3.78 (1H, dd, *J* = 10.8, 1.5 Hz) and *δ* 3.32 (1H, d, *J* = 10.8 Hz) which was not present in Compound **1**. These spectral features indicate that Compound **3** is betulin, in which the C‐28 methyl group of lupeol is oxidized to a primary alcohol (Figure 2), responsible for the new oxymethylene signals. Additional confirmation of the structure was obtained by comparing its proton NMR spectral information with previously published data for betulin [45].

The ^1^H NMR spectral data of Compound **4** had similar patterns as the spectrum of Compound 1 (Figure S4a). The difference included the presence of six singlet methyl protons signals instead of seven. The signal of H‐30 methyl protons at *δ* 1.68 of Compound **1** was absent in this compound. Additionally, there was the presence of an aldehyde proton signal at *δ* 9.51 (1H, s) which was not present in Compound **1**. The presence of an aldehyde group was also confirmed by ^13^C NMR signal at *δ* 195.14 (Figure S4b). The signal of olefinic methylene protons (H‐29) was found to be shifted to a more downfield region (*δ* 5.90 and 6.27) than that in Compound **1** indicating that the aldehyde group is attached directly to the olefinic group. All these spectral information suggested Compound **4** to be 3*β*‐hydroxylupenal. The compound′s structure was further confirmed by comparing its proton NMR spectroscopic data to previously published data for 3*β*‐hydroxylupenal [46].

The ^1^H NMR spectral data of the Compound **5** suggested the compound to be *β*‐sitosterol (Figure 2, Figure S5, and Table 2). Confirmation of the compound′s structure was obtained by comparing its proton NMR spectroscopic data with previously reported data for *β*‐sitosterol [47].

### 3.2. Acute Oral Toxicity Study

The crude methanolic extract did not produce mortality or overt signs of toxicity at the tested oral dose level during the observation period. No marked behavioral abnormalities or gross toxic manifestations were observed. These findings supported the use of 100–400 mg/kg as pharmacological test doses in the subsequent in vivo experiments.

### 3.3. In Vivo Analgesic Activity of *C. decandra* Methanolic Extract

#### 3.3.1. Effect of the Methanolic Extract on Acetic Acid–Induced Writhing

*C. decandra* leaves showed a marked and concentration‐dependent reduction in acetic acid–induced writhing and stretching reflexes in mice, with statistical significance (*p* < 0.001) as shown in Table 3. When administered at 200 and 400 mg/kg body weight, the methanol extract of *C. decandra* leaves resulted in 34.78% and 45.65% reductions in writhing reflex, respectively. In comparison, the standard medication diclofenac sodium, given at 10 mg/kg body weight, produced a 48.80% (*p* < 0.001) inhibition of the writhing reflex in the test subjects.

**Table 3:** Effect of *C. decandra* leaf methanolic extract on writhing induced by acetic acid.

Group	Dose (mg/kg)	No. of writhings in 30 min	Writhing inhibition (%)	Adjusted *p* value vs. control
Control	—	69.00 ± 1.5	—	—
Standard	10	35.33 ± 0.88	48.80	< 0.001
CDME	200	45.00 ± 1.03	34.78	< 0.001
CDME	400	37.50 ± 0.76	45.65	< 0.001

*Note:* Values expressed as mean ± SEM (*n* = 6).

Abbreviation: CDME = *Ceriops decandra* methanolic extract.

#### 3.3.2. Effect of the Methanolic Extract in the Formalin‐Induced Licking Test

The methanol extract exhibited analgesic properties during both the initial (0–5 min) and subsequent (15–30 min) stages of the formalin test as shown in Table 4. These phases are associated with neurogenic and inflammatory pain, respectively. The extract significantly blocked both neurogenic and inflammation‐induced pain at the test concentrations. It demonstrated greater effectiveness in suppressing pain caused by neurogenic factors compared to pain resulting from inflammation, exhibiting a performance similar to the standard drug diclofenac sodium. The pain blockade capacity of the extract increased with increasing its dose. The highest inhibition was shown by the dose 400 mg/kg in both phases (30.61% and 16.49%, respectively, *p* < 0.001).

**Table 4:** Effect of methanolic extract of *C. decandra* leaves on formalin‐induced pain.

Group	Dose (mg/kg)	Number of licks					
		0–5 min			15–30 min		
		Licks	% inhibition	Adjusted *p* value vs. control	Licks	% inhibition	Adjusted *p* value vs. control
Control	—	65.33 ± 1.41	—	—	131.33 ± 2.73	—	—
Standard	10	37.67 ± 0.88	42.34	< 0.001	86.50 ± 2.29	34.14	< 0.001
CDME	200	54.83 ± 1.78	16.07	0.000137	118.17 ± 2.54	10.02	0.002420
CDME	400	45.33 ± 1.54	30.61	< 0.001	109.67 ± 1.90	16.49	< 0.001

*Note:* Values expressed as mean ± SEM (*n* = 6).

#### 3.3.3. Effect of the Methanolic Extract in the Hot Plate Test

The hot plate test findings (Table 5) demonstrated that the *C. decandra* leaf methanol extract significantly extended the mice′s reaction time after 1 h. The increase in reaction time was proportional to the dose administered. This extension in reaction time peaked at 100.59% (*p* < 0.001) after 1.5 h with a 400 mg/kg dose. In comparison, diclofenac sodium at 10 mg/kg exhibited its peak protective effect of 198.46% (*p* < 0.001) after 1 h, whereas the extract at 400 mg/kg showed a 45.36% (*p* < 0.001) effect during the same period.

**Table 5:** Effect of *C. decandra* leaf methanolic extract on hot plate‐induced pain.

Group	Dose (mg/kg)	Latency period (h)								
		0 h	0.5 h	Adjusted *p* value vs. control	1 h	Adjusted *p* value vs. control	1.5 h	Adjusted *p* value vs. control	2 h	Adjusted *p* value vs. control
Control	—	5.78 ± 0.11	5.88 ± 0.11 (1.73)	—	5.73 ± 0.08 (−0.87)	—	5.78 ± 0.06 (0)	—	5.30 ± 0.18 (−8.30)	—
Standard	10	5.20 ± 0.07	13.77 ± 0.47 (164.80)	< 0.001	15.52 ± 0.32 (198.46)	< 0.001	13.52 ± 0.35 (160)	< 0.001	9.13 ± 0.29 (75.58)	< 0.001
CDME	200	5.15 ± 0.11	5.85 ± 0.14 (13.59)	0.99	6.70 ± 0.13 (30.10)	0.004	7.55 ± 0.23 (46.60)	< 0.001	7.85 ± 0.07 (52.43)	< 0.001
CDME	400	5.07 ± 0.11	6.18 ± 0.08 (21.89)	0.74	7.37 ± 0.07 (45.36)	< 0.001	10.17 ± 0.26 (100.59)	< 0.001	9.60 ± 0.19 (89.35)	< 0.001

*Note:* Values are expressed as mean ± SEM (*n* = 6) and are in seconds. The extract′s effectiveness in reducing pain caused by hot plate is expressed as a percentage of pain inhibition (%I) and is shown in parentheses.

#### 3.3.4. Effect of the Methanolic Extract in the Tail Immersion Test

The plant extract, when administered i.p. at 200 mg/kg, demonstrated a notable decrease in pain sensitivity (6.85%, *p* < 0.001) following a 0.5‐h latency period. This effect, observed during tail immersion in warm water, was found to be dose‐dependent (Table 6). The extract′s inhibitory effects were significant between 0.5 and 1 h after administration, peaking at 7.03% (*p* < 0.001) with a 400 mg/kg dose. Throughout the study, the extract consistently exhibited superior pain‐relieving properties compared to the standard drug, diclofenac sodium.

**Table 6:** Effect of *C. decandra* leaf methanolic extract on pain using tail immersion test.

Group	Dose (mg/kg)	Latency period (h)								
		0 h	0.5 h	Adjusted *p* value vs. control	1 h	Adjusted *p* value vs. control	1.5 h	Adjusted *p* value vs. control	2 h	Adjusted *p* value vs. control
Control	0	2.25 ± 0.04	2.39 ± 0.06 (6.22)	—	2.31 ± 0.04 (2.66)	—	2.39 ± 0.05 (6.22)	—	2.33 ± 0.05 (3.56)	—
Standard	10	4.16 ± 0.03	4.17 ± 0.02 (0.24)	< 0.001	4.15 ± 0.02 (−0.24)	< 0.001	4.12 ± 0.18 (−0.96)	< 0.001	4.01 ± 0.12 (−3.61)	< 0.001
CDME	200	2.92 ± 0.10	3.12 ± 0.04 (6.85)	< 0.001	3.02 ± 0.05 (3.42)	< 0.001	2.63 ± 0.08 (−9.93)	0.256	2.58 ± 0.08 (−11.64)	0.058
CDME	400	3.27 ± 0.02	3.50 ± 0.03 (7.03)	< 0.001	3.37 ± 0.02 (3.06)	< 0.001	3.37 ± 0.03 (3.06)	< 0.001	3.25 ± 0.04 (−6.54)	< 0.001

*Note:* Values are expressed as mean ± SEM (*n* = 6) and are in seconds. Values in parentheses indicate the percentage change in latency relative to baseline. Negative values indicate shorter withdrawal times than baseline.

#### 3.3.5. Effect of the Methanolic Extract in the Tail Clip Test

In the tail clip test, the initial reaction time in control was noted as 6.81 s. Following drug administration, a notable rise in reaction time was detected for a period of 30 min. The leaf extract at doses of 200 and 400 mg/kg body weight was able to inhibit the pain by 49.63% (*p* < 0.001) and 96.92% (*p* < 0.001), respectively. The standard diclofenac sodium (10 mg/kg) exhibited a marked (*p* < 0.001) increase in retention time of 17.33 s equivalent to 154.48% pain inhibition (Table 7).

**Table 7:** Impact of methanolic extract of *C. decandra* leaves on pain using tail clip test.

Group	Dose (mg/kg)	Reaction time in seconds (mean ± SEM)	% inhibition	Adjusted *p* value vs. control
Control	—	6.81 ± 0.14	—	—
Standard	10	17.33 ± 0.22	154.48	< 0.001
CDME	200	10.19 ± 0.14	49.63	< 0.001
CDME	400	13.41 ± 0.10	96.92	< 0.001

### 3.4. In Vivo Anti‐Inflammatory Activity of *C. decandra* Methanolic Extract

Following carrageenan injection, mice experienced a gradual increase in paw edema, peaking after 1 h. The *C. decandra* leaf methanolic extract significantly mitigated this carrageenan‐induced paw swelling in mice across all tested doses (*p* < 0.001). The 200 mg/kg body weight dose exhibited more potent edema inhibition compared to the 100 mg/kg body weight dose when contrasted with the control group (Table 8). After 4 h postcarrageenan injection, the extract demonstrated anti‐inflammatory effects of 18.62% (*p* < 0.001) and 29.81% (*p* < 0.001) at doses of 100 and 200 mg/kg body weight, respectively.

**Table 8:** Impact of methanolic extract of *C. decandra* leaves on carrageenan‐induced inflammation.

Group	Dose (mg/kg)	Mean paw volume (mm) ± SEM							
		First hour	Adjusted *p* value vs. control	Second hour	Adjusted *p* value vs. control	Third hour	Adjusted *p* value vs. control	Fourth hour	Adjusted *p* value vs. control
Control	—	4.54 ± 0.15	—	4.94 ± 0.13	—	4.83 ± 0.18	—	4.35 ± 0.04	—
Standard	10	3.75 ± 0.16 (17.4)	< 0.001	3.50 ± 0.13 (29.15)	< 0.001	3.13 ± 0.04 (35.20)	< 0.001	2.44 ± 0.16 (43.91)	< 0.001
CDME	100	4.34 ± 0.06 (4.41)	0.493	4.26 ± 0.04 (13.77)	< 0.001	4.10 ± 0.04 (15.11)	< 0.001	3.54 ± 0.14 (18.62)	< 0.001
CDME	200	4.14 ± 0.02 (8.81)	0.060	4.07 ± 0.02 (17.61)	< 0.001	3.39 ± 0.02 (29.81)	< 0.001	3.13 ± 0.04 (28.05)	< 0.001

*Note:* % inhibition of inflammatory effects (%I) are given in parenthesis.

### 3.5. Molecular Docking Analysis of Isolated Compounds Against Pain‐ and Inflammation‐Related Targets

The binding affinities of the best conformations of the ligands against the selected targets are presented in Table 9. All the phytochemical compounds showed very prominent binding affinities against mu and COX‐2 receptors. 3*β*‐hydroxylupenal showed the highest binding affinity (−7.7 kcal/mol) against the mu receptor which is even higher than the standard diclofenac sodium (−7.5 kcal/mol), indicating that it has the potential to exhibit better central analgesic activity than diclofenac sodium. Its binding affinities against COX‐1 and COX‐2 receptors were 1.1 and −6.5 kcal/mol, respectively, which suggest that it can also act as a selective COX‐2 inhibitor to exert peripheral analgesia. It was discovered to interact with the mu receptor through various molecular connections involving specific amino acid residues. These interactions included alkyl bonds with Val143, Ile144, Cys217, Val236, and Val300, as well as pi‐alkyl bonds with His54, Trp133, Tyr148, His297, and Trp318. It bonded to the COX‐2 receptor via conventional hydrogen bonds with Phe580 and Ser581.

**Table 9:** Receptor binding strengths of isolated compounds and the standard against mu, COX‐1, and COX‐2 receptors.

Compound	Binding affinity (kcal/mol)		
	Central analgesic	Peripheral analgesic	
	Mu	COX‐1	COX‐2
Diclofenac sodium	−7.5	−8.2	−6.8
Lupeol	−6.9	−6.3	−6.9
3*β*‐*E*‐coumaroyllupeol	−6.5	4.8	−8.2
Betulin	−6.9	3.1	−6.7
3*β*‐hydroxylupenal	−7.7	1.1	−6.5

3*β*‐*E*‐coumaroyllupeol exhibited a significantly stronger binding affinity (−8.2 kcal/mol) to the COX‐2 receptor compared to the standard diclofenac sodium (−6.8 kcal/mol). It also demonstrated low binding affinity toward COX‐1 and prominent binding affinity toward the mu receptor, indicating its potential to act both as a central and selective peripheral analgesic agent. It bonded to the COX‐2 receptor via the amino acid residues: pi‐pi T‐shaped (His95) and pi‐alkyl (His351 and Pro514). It was found to bind with the mu receptor via the amino acids: conventional hydrogen bond (His297), Alkyl (Val143, Met151, Ile296, Val300, Ile322), and Pi‐alkyl (His54, Tyr75, Trp133, Tyr148, His319, Leu232, Lys233, and Val300).

Betulin displayed prominent binding affinity toward Mu and COX‐2 but low affinity toward COX‐1 receptor. On the other hand, lupeol showed prominent binding affinity toward each of the receptors. The noncovalent interactions of the ligands with the targets are presented in Table 10 and Figures 3, 4, and 5. This is the first report of molecular docking of 3*β*‐*E*‐coumaroyllupeol and 3*β*‐hydroxylupenal with these targets. However, these in silico findings should be interpreted as mechanistic support only, since the isolated compounds were not evaluated individually in vivo in the present study.

**Table 10:** Noncovalent interactions of isolated compounds and standard with mu, COX‐1, and COX‐2 receptors.

Receptor	Compound	Bond type	Amino acids
Mu	Diclofenac sodium	Pi‐donor hydrogen bond	His54
		Pi‐alkyl	Ile144, Ile322
	Lupeol	Alkyl	Ile144, Met151, Cys217, Val236, Ile296, Val300, Ile322
		Pi‐alkyl	His54, Trp133, Tyr148, Trp293, His297
	3*β*‐*E*‐coumaroyllupeol	Conventional hydrogen bond	His297
		Alkyl	Val143, Met151, Ile296, Val300, Ile322
		Pi‐alkyl	His54, Tyr75, Trp133, Tyr148, His319, Leu232, Lys233, Val300
	Betulin	Carbon hydrogen bond	His54
		Alkyl	Ile144, Met151, Cys217, Val236, Ile296, Val300, Ile322
		Pi‐alkyl	Trp133, Tyr148, Trp293, His297, Tyr326
	3*β*‐hydroxylupenal	Alkyl	Val143, Ile144, Cys217, Val236, Val300
		Pi‐alkyl	His54, Trp133, Tyr148, His297, Trp318

COX‐1	Diclofenac sodium	Amide‐Pi stacked	Ala202, Gln203
		Carbon hydrogen bond	His388
		Pi‐Pi T‐shaped	His388
		Conventional hydrogen bond	His388, Met391
		Pi‐alkyl	Ala202
	Lupeol	Carbon hydrogen bond	Glu346
		Alkyl	Arg581
		Pi‐alkyl	Phe356
	3*β*‐*E*‐coumaroyllupeol	Conventional hydrogen bond	Leu112, Val116, Tyr348, Leu352, Tyr355, Phe381, Tyr385, Trp387, Ile523, Ala527, Leu531
		Pi‐sigma	Leu93
		Pi‐alkyl	Leu112, Val116, Tyr348, Tyr355, Leu357, Phe381, Tyr385, Trp387, Phe518
		Alkyl	Val116, Val349, Leu352, Leu359, Met522, Ile523, Ala527, Leu531
		Carbon hydrogen bond	Leu93
	Betulin	Conventional hydrogen bond	Tyr348, Phe381, Leu352, Tyr385, Trp387, Ile523, Ala527, Leu531
		Carbon hydrogen bond	Met522
		Alkyl	Val116, Val349, Leu352, Leu359, Ile523, Ala527, Leu531
		Pi‐alkyl	Tyr348, Tyr355, Phe381, Tyr385, Trp387, Phe518
	3*β*‐hydroxylupenal	Alkyl	Val116, Val349, Leu352, Leu359, Leu384, Met522, Ala527, Leu531
		Sulfur‐X	Met113
		Conventional hydrogen bond	Met113
		Pi‐alkyl	Tyr355, Trp387, Phe518

COX‐2	Diclofenac sodium	Conventional hydrogen bond	Arg120, Tyr355
		Pi‐Sigma	Val89
		Pi‐Pi T‐shaped	Tyr115
		Pi‐alkyl	Ile112
	Lupeol	Pi‐alkyl	His351, His356
	3*β*‐*E*‐coumaroyllupeol	Pi‐alkyl	His351, Pro514
		Pi‐Pi T‐shaped	His95
	Betulin	Conventional hydrogen bond	Gly354
		Pi‐alkyl	His356
	3*β*‐hydroxylupenal	Conventional hydrogen bond	Phe580, Ser581

Figure 33D image of molecular docking model (left) and 2D image of noncovalent interactions (right) of (A) diclofenac sodium, (B) lupeol, (C) 3*β*‐*E*‐coumaroyllupeol, (D) betulin, and (E) 3*β*‐hydroxylupenal at the binding site of mu receptor.

**Figure:**
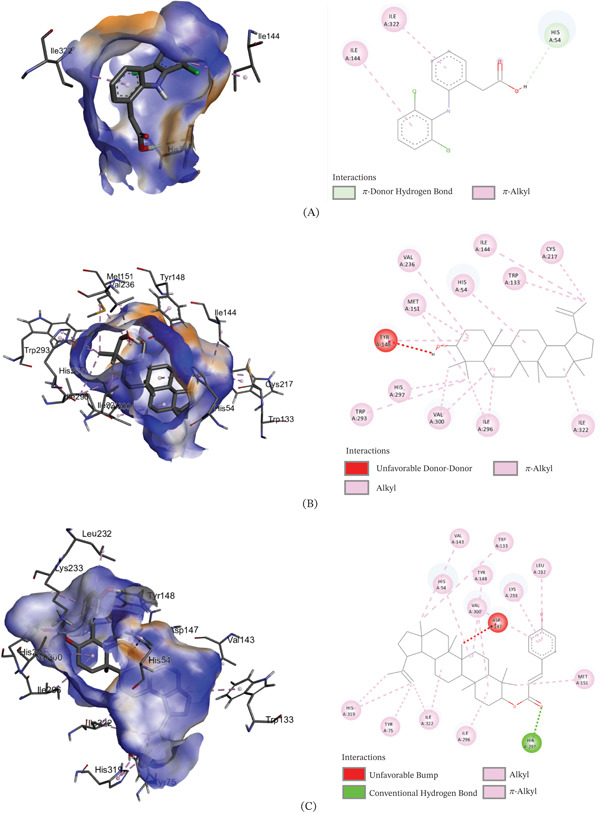
(A)

**Figure:**
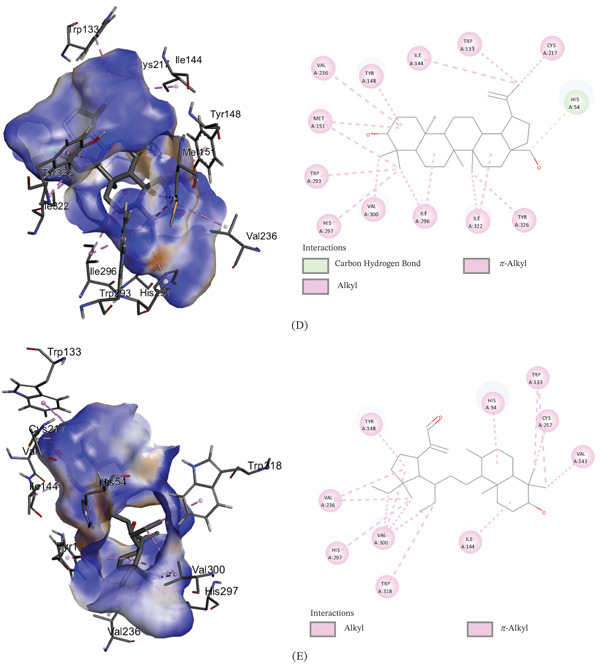
(B)

Figure 43D image of molecular docking model (left) and 2D image of noncovalent interactions (right) of (A) diclofenac sodium, (B) lupeol, (C) 3*β*‐*E*‐coumaroyllupeol, (D) betulin, and (E) 3*β*‐hydroxylupenal at the binding site of COX‐1 receptor.

**Figure:**
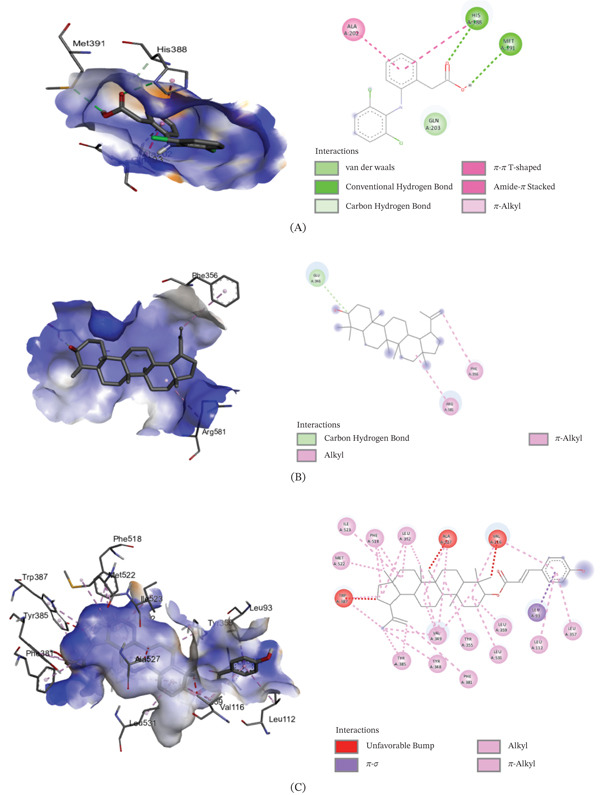
(A)

**Figure:**
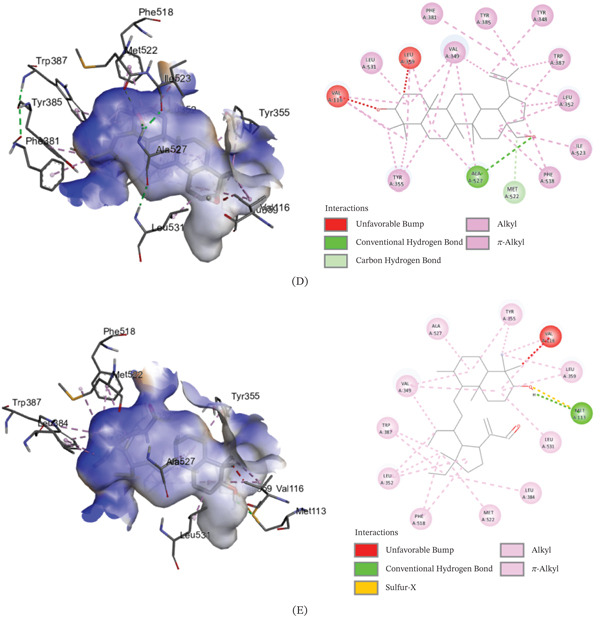
(B)

Figure 53D image of molecular docking model (left) and 2D image of noncovalent interactions of (A) diclofenac sodium, (B) lupeol, (C) 3*β*‐*E*‐coumaroyllupeol, (D) betulin, and (E) 3*β*‐hydroxylupenal at the binding site of COX‐2 receptor.

**Figure:**
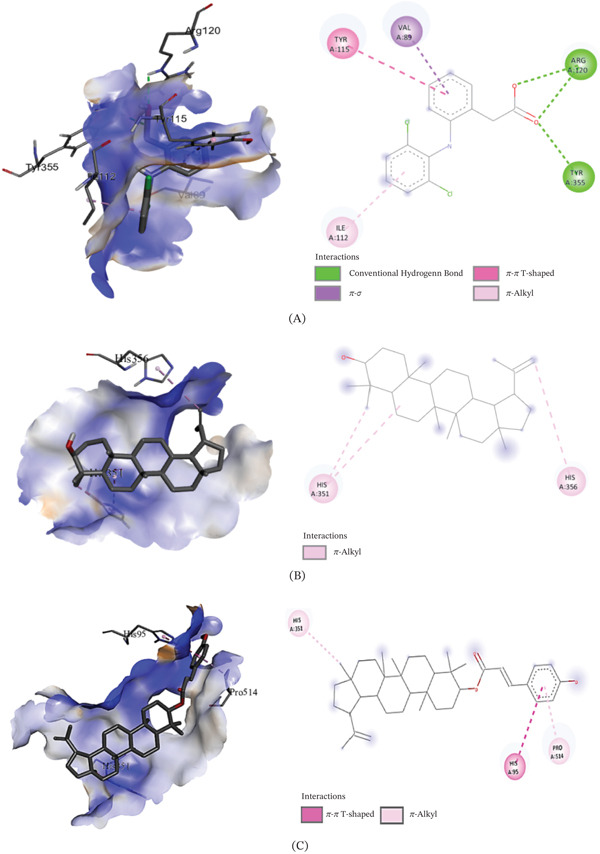
(A)

**Figure:**
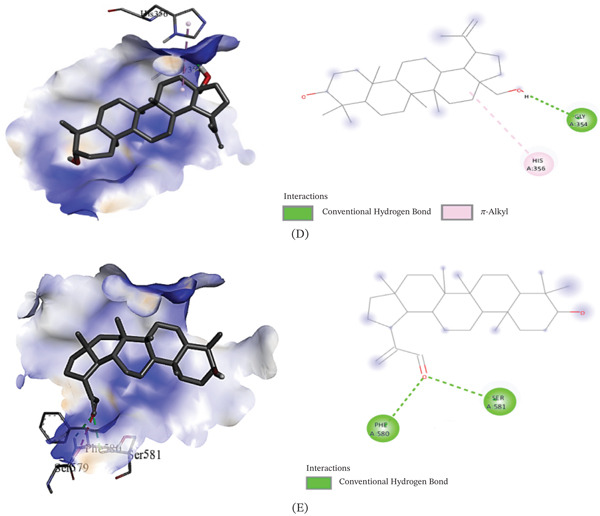
(B)

### 3.6. In Silico ADMET Prediction of the Isolated Compounds

The results of ADMET prediction are presented in Table 11. Lupeol, betulin, and 3*β*‐hydroxylupenal each violated only one criterion (MW) of Lipinski′s guideline. They are likely to have good oral bioavailability. But 3*β*‐*E*‐coumaroyllupeol violated two criteria (MW and logP) and is predicted to have poor oral bioavailability. All the compounds were predicted to be safe from hepatotoxicity, carcinogenicity, and cytotoxicity. This is the first report of ADMET properties of 3*β*‐*E*‐coumaroyllupeol and 3*β*‐hydroxylupenal by computational methods.

**Table 11:** Predicted ADMET parameters of isolated phytochemicals.

Compound	MW (Da)	nHBD	nHBA	logP	OBS	nLV	Toxicity		
							HT	CG	CT
Lupeol	426.72	1	1	7.26	0.55	1	Inactive	Inactive	Inactive
3*β*‐*E*‐coumaroyllupeol	572.86	1	3	8.75	0.17	2	Inactive	Inactive	Inactive
Betulin	442.72	2	2	6.36	0.55	1	Inactive	Inactive	Inactive
3*β*‐hydroxylupenal	440.70	1	2	6.47	0.55	1	Inactive	Inactive	Inactive

Abbreviations: CG = carcinogenicity, CT = cytotoxicity, HT = hepatotoxicity, logP = lipophilicity, MW = molecular weight, nHBA = no. of hydrogen bond acceptor, nHBD = no. of hydrogen bond donor, nLV = no. of Lipinski violation, OBS = oral bioavailability score.

### 3.7. PASS‐Based Prediction of Pharmacological Activities of the Isolated Compounds

The isolated compounds were studied by PASS for their analgesic activities. All of them showed greater potential activity (Pa) than potential inactivity (Pi) (Table 12). Lupeol showed the highest Pa score (0.726) for analgesic activity followed by 3*β*‐hydroxylupenal (0.720) and betulin (0.691). 3*β*‐E‐coumaroyllupeol showed the lowest score (0.575) among the compounds studied. Some highly possible pharmacological activities (Pa > 0.9) of these compounds were also predicted and shown in Table 12. This is the first report of predicted biological activities of 3*β*‐*E*‐coumaroyllupeol and 3*β*‐hydroxylupenal by in silico method.

**Table 12:** Pass prediction of the isolated compounds for analgesic activity.

Compounds	Pass prediction of analgesic activity		Other predicted activities with Pa > 0.9
	Pa	Pi	
Lupeol	0.726	0.003	Caspase 3 stimulant, antineoplastic, transcription factor NF kappa B stimulant, transcription factor stimulant, hepatoprotectant
3ß‐E‐coumaroyllupeol	0.575	0.010	Hepatoprotectant, antiprotozoal (Leishmania), caspase 3 stimulant, chemopreventive, antineoplastic, mucomembranous protector
Betulin	0.691	0.004	Caspase 3 stimulant, antineoplastic (melanoma), hepatoprotectant, antineoplastic, transcription factor stimulant, transcription factor NF kappa B stimulant
3*β*‐hydroxylupenal	0.720	0.003	Transcription factor stimulant, apoptosis agonist, antineoplastic, caspase 3 stimulant, transcription factor NF kappa B stimulant

## 4. Discussion

The present study expands the phytochemical profile of Bangladeshi‐origin *C. decandra* leaves and links that chemistry to a preliminary, coherent pharmacological signal. Five compounds were isolated from the methanolic leaf extract, namely lupeol (**1**), 3*β*‐E‐coumaroyllupeol (**2**), betulin (**3**), 3*β*‐hydroxylupenal (**4**), and *β*‐sitosterol (**5**). Notably, 3*β*‐hydroxylupenal is reported here for the first time from *C. decandra*, whereas Compounds **1–3** are newly reported from Bangladeshi‐origin leaves, although they have been described previously from this plant in other geographic contexts. The findings indicate that the leaf extract is rich in lupane‐type triterpenoids and related lipophilic constituents. This pattern is pharmacologically plausible because both lupeol‐ and betulin‐type scaffolds have repeatedly been associated with anti‐inflammatory activity in recent preclinical literature [48, 49]. In that sense, the phytochemical results are not merely descriptive. They provide a reasonable chemical context for the biological effects observed in the present work.

The in vivo data suggest a mixed analgesic profile, with a stronger peripheral component and a more modest, but still detectable, central component. The clearest signal came from the acetic acid–induced writhing assay, where the extract reduced abdominal constrictions in a dose‐dependent fashion and the higher dose (400 mg/kg) approached the effect of diclofenac. This pattern supports a peripheral antinociceptive action. Acetic acid–induced writhing is widely used to reflect peripheral nociception driven by the release of prostaglandins, particularly PGE2, PGF2a, and lipoxygenase‐derived eicosanoids [50, 51]. In this context, the marked reduction in writhing observed in the present study suggests that the extract may interfere with mediator generation upstream or downstream of prostaglandin production. This interpretation is also consistent with the docking results, which showed favorable predicted interactions of several isolated constituents with COX‐1 and COX‐2. The writhing data support the view that inhibition of inflammatory mediator signaling is likely to be one of the main contributors to the extract′s analgesic effect.

The formalin test further refined this interpretation. The formalin model is commonly interpreted as a biphasic assay in which the first phase reflects the direct activation of nociceptive afferents, whereas the second phase involves inflammatory processes together with central sensitization [52, 53]. The extract reduced nociceptive behavior in both phases of the test, although the effect was more pronounced in the early neurogenic phase than in the later inflammatory phase. This suggests a mixed analgesic profile rather than a purely peripheral mechanism. At the same time, the more modest inhibition in the late phase suggests that the anti‐inflammatory contribution, while present, may be less prominent than the central antinociceptive component under this model.

Acetic acid writhing and the inflammatory phase of the formalin test are commonly used to capture peripheral nociception, whereas hot plate and tail immersion assays are more informative for centrally acting or opioid‐responsive analgesic effects [51, 54]. The increases in reaction latency observed in the hot plate and tail immersion tests were lower than those produced by the standard drug, but they remain biologically relevant for several reasons. First, both assays are indicative of central nociceptive processing, and the significant prolongation of latency produced by the extract suggests that *C. decandra* is not acting exclusively through peripheral pathways. Second, the activity showed a dose‐responsive trend, particularly at 400 mg/kg, which supports a genuine pharmacological effect rather than random variation. Third, in phytopharmacological studies, crude extracts commonly produce smaller responses than reference analgesics because the active constituents are present at lower effective concentrations within a complex mixture. Therefore, the significant and dose‐dependent latency prolongation observed here still supports meaningful central antinociceptive activity, with possible involvement of opioid‐responsive pathways.

The findings of this study also fit reasonably well with the limited literature already available on *C. decandra* leaves. Uddin et al. (2005) reported dose‐dependent antinociceptive activity of the ethanol extract of *C. decandra* leaf and pneumatophore in the acetic acid writhing model, showing that pain‐relieving potential had already been hinted at in the leaf material [17]. More recently, Nahar et al. (2023) described the anti‐inflammatory and anti‐ulcer activity of the methanolic leaf extract and isolated lupeol from the leaves, again pointing to the leaf as a pharmacologically active organ [55]. A separate recent study by Dey et al. further showed that *C. decandra* leaves were active in an allergic inflammation model and reported no overt acute toxicity up to 3000 mg/kg in mice [56]. Against that background, the present study adds two useful advances. First, it extends the biological profile of the leaves beyond a single nociceptive assay by using multiple pain models that collectively allow a more nuanced interpretation of peripheral and central responses. Second, it broadens the chemical evidence by identifying additional triterpenoid constituents that may contribute to the observed activity.

The anti‐inflammatory result deserves separate emphasis. In the carrageenan‐induced paw edema model, the extract produced its strongest inhibition at 3 h postcarrageenan, where the 200 mg/kg dose reached 29.81% inhibition. That timing is important because later carrageenan responses are commonly linked to prostaglandin‐rich inflammatory signaling rather than only to the earliest vasoactive events [51, 57]. Another potential mechanism of action could involve inhibiting the release of histamine, serotonin, and bradykinin, which are involved in both the early and late stages of carrageenan‐induced inflammation [58]. This interpretation is also consistent with the analgesic results, since suppression of inflammatory signaling would be expected to reduce writhing behavior and the inflammatory phase of formalin nociception. Thus, the carrageenan results complement the writhing data and strengthen the overall impression that modulation of inflammatory mediator pathways is one of the major mechanisms underlying the extract′s pharmacological activity. At the same time, the extract remained less active than the reference drug, which is not surprising for an unfractionated plant preparation. The findings suggest that *C. decandra* leaves do show anti‐inflammatory activity at the extract level, but the response is more consistent with an early‐stage crude‐extract study than with a purified anti‐inflammatory agent. Because the carrageenan paw edema model has historically been more widely standardized in rats, the present anti‐inflammatory findings should be interpreted within the context of a validated murine model, recognizing that the magnitude and time course of edema can vary across mouse strains and between mice and rats [31, 32].

Integrated experimental and in silico approaches are increasingly used in preclinical pharmacology to generate mechanistic hypotheses [59]. In the molecular docking analysis, 3*β*‐hydroxylupenal showed the most favorable predicted binding affinity toward the *μ*‐opioid receptor (−7.7 kcal/mol), slightly exceeding that of diclofenac sodium (−7.5 kcal/mol). It also showed a more favorable predicted interaction with COX‐2 (−6.6 kcal/mol) than with COX‐1 (−1.1 kcal/mol). Similarly, 3*β*‐E‐coumaroyllupeol showed a favorable predicted interaction with COX‐2 (−8.2 kcal/mol), whereas its COX‐1 score was positive (+4.8 kcal/mol), indicating an unfavorable predicted interaction in the latter model. This pattern suggests a possible COX‐2 preference at the in silico level. Lupeol and betulin also showed moderate predicted interactions with the *μ*‐opioid receptor and COX‐2, whereas lupeol exhibited the most favorable COX‐1 docking score among the tested compounds (−6.3 kcal/mol). These in silico findings suggest that the isolated triterpenoids may interact with both central and peripheral pain‐related targets, with some compounds showing a more favorable predicted preference for COX‐2. These favorable docking scores of the isolated compounds suggest plausible interactions with pain and inflammation‐related molecular targets and may help explain the analgesic and anti‐inflammatory effects observed with the crude extract. However, these results should be interpreted cautiously because the isolated compounds were not evaluated individually in vivo, and no functional receptor‐ or enzyme‐level validation was performed. Therefore, the present study cannot establish a direct one‐to‐one mechanistic relationship between any single isolated constituent and the observed pharmacological effects of CDME. Similarly, stronger predicted binding affinity should not be interpreted as direct evidence of prolonged receptor occupancy or toxicity. Docking scores provide only a simplified estimate of ligand–target interaction, whereas residence time depends on binding kinetics, particularly the dissociation rate, and toxicity is influenced by additional factors including selectivity, off‐target interactions, pharmacokinetics, metabolism, and exposure. Crude plant extracts represent complex mixtures in which activity may arise from the combined effects of multiple constituents, including additive or synergistic interactions, rather than from a single compound alone. Docking reflects only target‐binding plausibility and does not account for in vivo exposure, bioavailability, metabolism, tissue distribution, or actual receptor activation/inhibition. Individual in vivo testing of the isolated compounds was beyond the scope of the present work and was also limited by the small quantity of purified compounds available. Accordingly, the docking results are best interpreted as hypothesis‐generating evidence that helps prioritize compounds for future pharmacological and mechanistic validation.

The ADMET findings offer an initial indication of whether the isolated compounds possess physicochemical and safety‐related features that justify further pharmacological interest. It is often done in the drug discovery process to determine if the candidates fulfill Lipinski′s guideline, also referred to as the Rule of Five. This set of criteria is utilized to assess the drug‐likeness and potential oral bioavailability of small molecules. According to this guideline, a compound is more likely to be orally bioavailable if it does not violate more than one of the following requirements: molecular mass < 500 Da, hydrogen bond donors (HBDs) < 5, hydrogen bond acceptors (HBAs) < 10, and lipophilicity value logP < 5 [60]. In this study, the predicted profiles were moderately encouraging, although high lipophilicity emerged as the most consistent limitation. Most of the isolated constituents violated only the logP criterion of Lipinski′s rule, suggesting that limited solubility may be a greater concern than general lack of drug‐likeness. In contrast, 3*β*‐E‐coumaroyllupeol showed a less favorable profile because it exceeded both the molecular weight and lipophilicity thresholds. Except for this compound, the isolated constituents showed a bioavailability score of 0.55, which supports their consideration as early lead‐like molecules rather than optimized drug candidates. None of the studied compounds showed predicted hepatotoxicity, carcinogenicity, or cytotoxicity. These results should not be interpreted as proof of safety or oral suitability, but they do suggest that several of the isolated triterpenoids may be worth pursuing further, particularly if future work addresses formulation and exposure‐related limitations.

In silico PASS study refers to the computational prediction of biological activity spectra for chemical substances. It uses a set of structural and property‐based features to predict biological activities and thus contributes to the early identification of promising compounds in the drug discovery process [61]. The PASS prediction system assigns values for Pa and Pi to each predicted activity, ranging from 0.000 to 1.000. For any given compound, only activities where Pa exceeds Pi are deemed potential. Activities with Pa greater than 0.7 have a high likelihood of yielding positive experimental pharmacological results. When Pa falls between 0.5 and 0.7, the chances of experimental pharmacological action are reduced. Pa values below 0.5 indicate a lower likelihood of experimental confirmation, but they may also suggest the potential for discovering a new compound [41]. In the current study, the PASS results were broadly consistent with the overall pharmacological direction of the study and helped to prioritize the isolated constituents for future work. All compounds showed Pa values above 0.5 for analgesic activity, indicating a fair to high probability of demonstrating such effects experimentally. This pattern aligns reasonably well with the in vivo findings and the docking results, both of which suggest that the isolated constituents may contribute to the analgesic profile of the crude extract. In addition, all compounds showed very high predicted anticancer potential, with Pa values above 0.9. While these predictions are useful at the screening stage, they should be interpreted with caution. PASS does not confirm biological activity in a living system, rather it serves as a supportive computational tool that helps identify compounds deserving closer experimental evaluation. [62].

Nevertheless, this study has some limitations. The isolated compounds were not tested individually in vivo, and the docking predictions were not supported by functional receptor‐ or enzyme‐level validation. As a result, the present findings cannot establish a direct one‐to‐one mechanistic relationship between any single isolated constituent and the observed analgesic and anti‐inflammatory effects of the crude extract. Future studies should therefore evaluate purified compounds, particularly 3*β*‐E‐coumaroyllupeol and 3*β*‐hydroxylupenal, in dose‐controlled animal models, together with receptor‐binding, antagonism, or enzyme‐inhibition assays, to verify their specific pharmacological contributions. Accordingly, dedicated kinetic, selectivity, and toxicological studies will be required before any conclusion can be drawn regarding the safety implications of these compounds. Because *C. decandra* is a “near threatened” mangrove species, future pharmacological development should be accompanied by sustainable sourcing strategies, including nondestructive leaf harvesting and reduced dependence on repeated wild collection.

## 5. Conclusion

This study provides a phytochemical and preclinical pharmacological investigation of Bangladeshi‐origin *C. decandra* leaves. Phytochemical isolation led to the identification of five compounds, including the first report of 3*β*‐hydroxylupenal from this species and the first isolation of three triterpenoids from its Bangladeshi leaves. At the extract level, the methanolic leaf extract produced significant analgesic effects in multiple murine models and also demonstrated anti‐inflammatory activity in the carrageenan‐induced paw edema assay, supporting the traditional relevance of this mangrove species in pain‐ and inflammation‐related conditions. In parallel, the molecular docking analysis suggested that several isolated constituents, particularly 3*β*‐hydroxylupenal and 3*β*‐E‐coumaroyllupeol, may plausibly interact with pain‐ and inflammation‐associated targets, including the *μ*‐opioid receptor, COX‐1, and COX‐2. From a practical perspective, these findings help prioritize *C. decandra* leaves as a pharmacologically relevant mangrove resource and identify 3*β*‐hydroxylupenal and 3*β*‐E‐coumaroyllupeol as candidate constituents for future bioactivity‐guided and mechanism‐oriented studies.

However, the findings should be interpreted with caution. The in vivo assays were conducted using the crude methanolic extract, whereas the isolated compounds were assessed only through in silico methods. Therefore, the present work does not establish a direct mechanistic contribution of any individual constituent to the observed biological effects. In addition, docking results indicate only target‐binding plausibility and were not supported by functional receptor‐ or enzyme‐level validation. As a result, this study should be viewed as an initial preclinical and hypothesis‐generating investigation rather than definitive proof of therapeutic utility. Future studies should focus on the individual in vivo evaluation of purified compounds, detailed mechanistic validation, and dose–response characterization to clarify the specific contributors and pathways underlying the observed analgesic and anti‐inflammatory activities.

## Author Contributions

**Asef Raj:** analysis, visualization, software, writing – main manuscript, writing – review and editing. **Sania Ashrafi**: validation. **Md. Sakhawat Hossain**: analysis. **Md. Saiful Islam**: supervision and analysis. **Muhammad Abdullah Al-Mansur**: analysis. **Abu Bin Ihsan**: writing – review and editing. **Md. Ruhul Kuddus**: writing – review and editing. **Firoj Ahmed**: conceptualization, project administration, resources, supervision, funding acquisition.

## Funding

This study was supported by the University of Dhaka, 10.13039/501100006523, Centennial Research Grant‐2021 (Reg/Ad‐3/47852).

## Conflicts of Interest

The authors declare no conflicts of interest.

## Supporting information


**Supporting Information** Additional supporting information can be found online in the Supporting Information section. Figure S1: ^1^H NMR spectrum of Compound **1** (lupeol). Figure S2: ^1^H NMR spectrum of Compound **2** (3*β*‐*E*‐coumaroyllupeol). Figure S3: ^1^H NMR spectrum of Compound **3** (betulin). Figure S4a: ^1^H NMR spectrum of Compound **4** (3*β*‐hydroxylupenal). Figure S4b: ^13^C NMR spectrum of Compound **4** (3*β*‐hydroxylupenal). Figure S5: ^1^H NMR spectrum of Compound **5** (*β*‐sitosterol).

## Data Availability

The data that support the findings of this study are available on request from the corresponding author. The data are not publicly available due to privacy or ethical restrictions.
