# Supplementary material for: Isolation of 3β‐Hydroxylupenal From Ceriops decandra Leaves and Evaluation of In Vivo Analgesic and Anti‐Inflammatory Effects Supported by Molecular Docking
Source: Biomed Res Int. 2026 May 18;2026:4730586. doi: 10.1155/bmri/4730586 (PMC13181330; doi:10.1155/bmri/4730586)
Supplement: Supplementary file 1 — Supporting Information Additional supporting information can be found online in the Supporting Information section. Figure S1: 1H NMR spectrum of Compound 1 (lupeol). Figure S2: 1H NMR spectrum of Compound 2 (3β‐E‐coumaroyllupeol). Figure S3: 1H NMR spectrum of Compound 3 (betulin). Figure S4a: 1H NMR spectrum of Compound 4 (3β‐hydroxylupenal). Figure S4b: 13C NMR spectrum of Compound 4 (3β‐hydroxylupenal). Figure S5: 1H NMR spectrum of Compound 5 (β‐sitosterol). [file BMRI-2026-4730586-s001.docx]

**Supplementary Data**


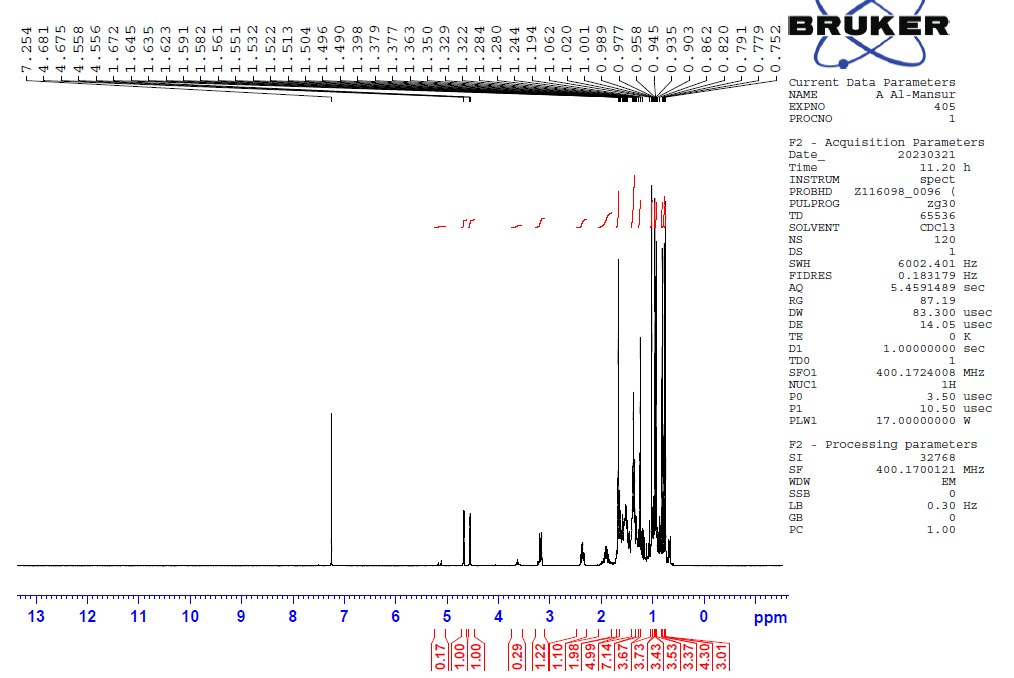


Figure S1: ^1^H NMR spectrum of compound **1** (Lupeol)


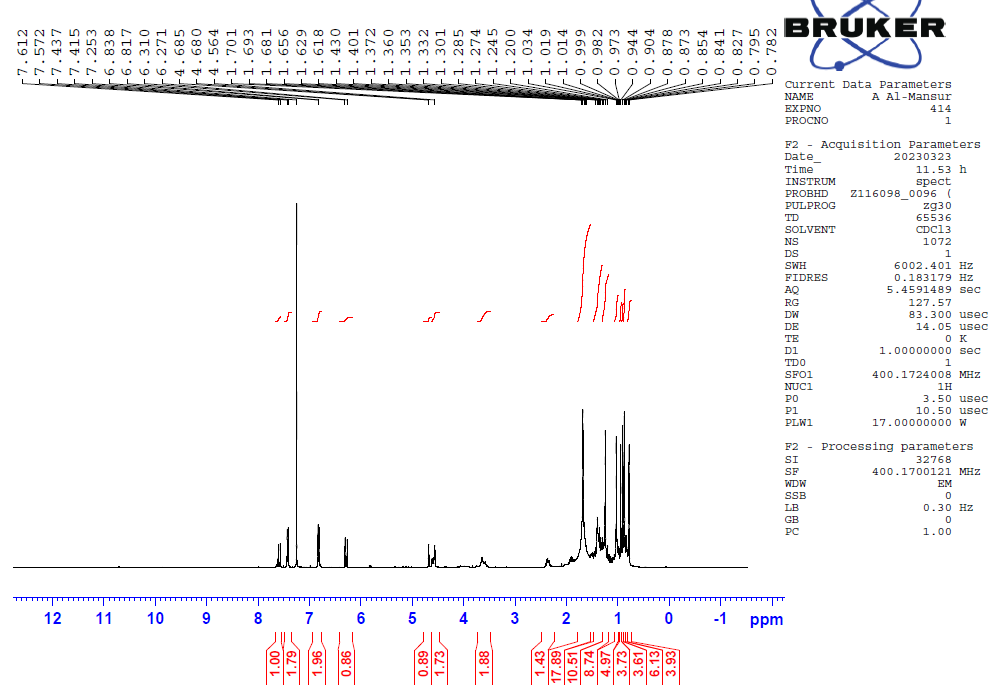


Figure S2: ^1^H NMR spectrum of compound **2** (3β-*E*-coumaroyllupeol)


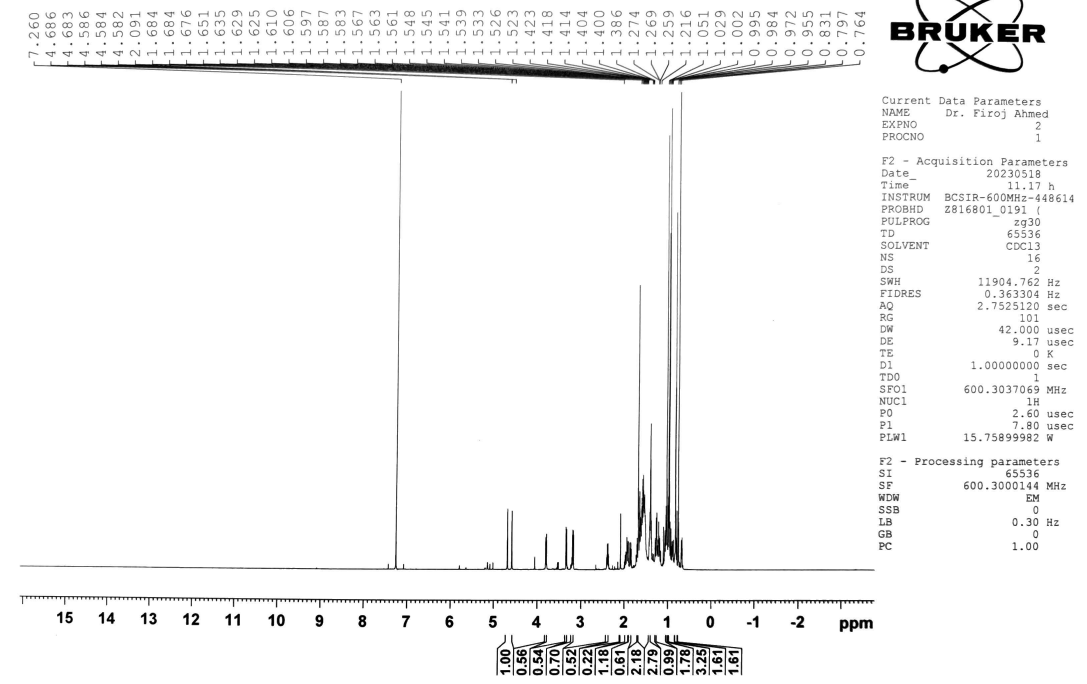


Figure S3: ^1^H NMR spectrum of compound **3** (Betulin)


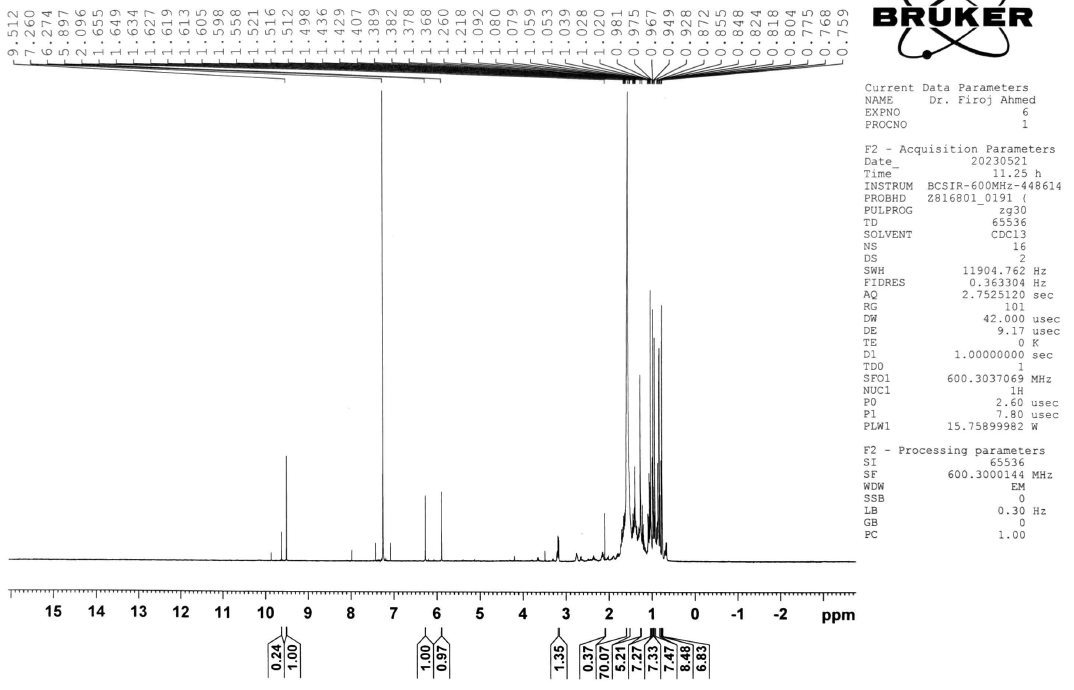


Figure S4a: ^1^H NMR spectrum of compound **4** (3β-hydroxylupenal)


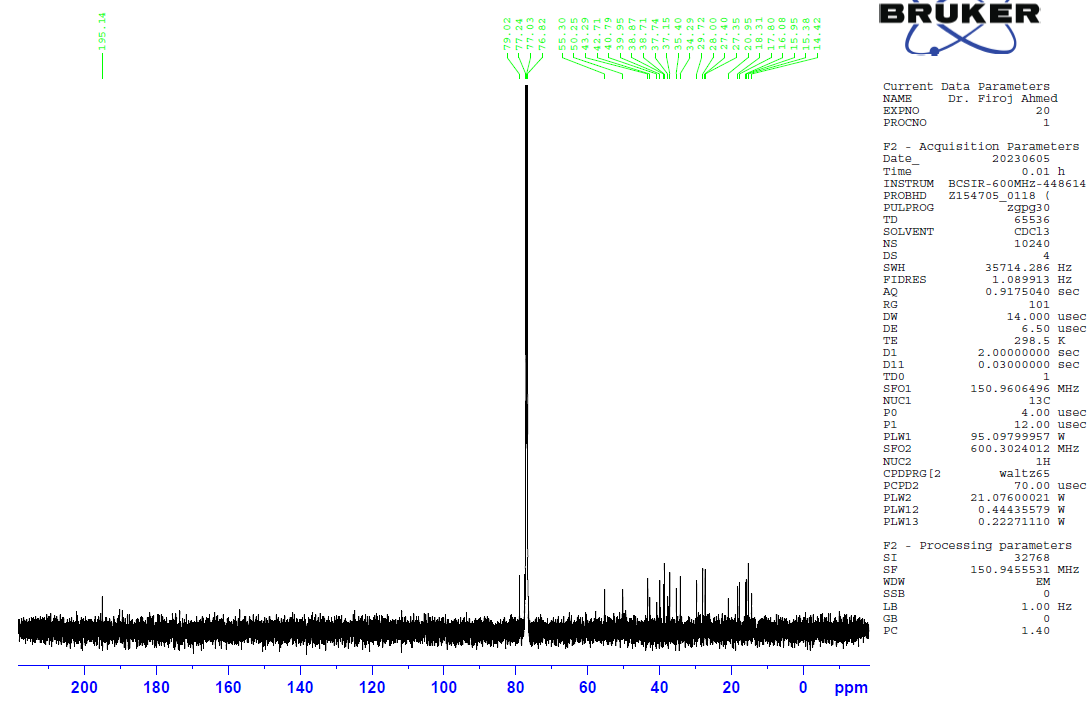


Figure S4b: ^13^C NMR spectrum of compound **4** (3β-hydroxylupenal)


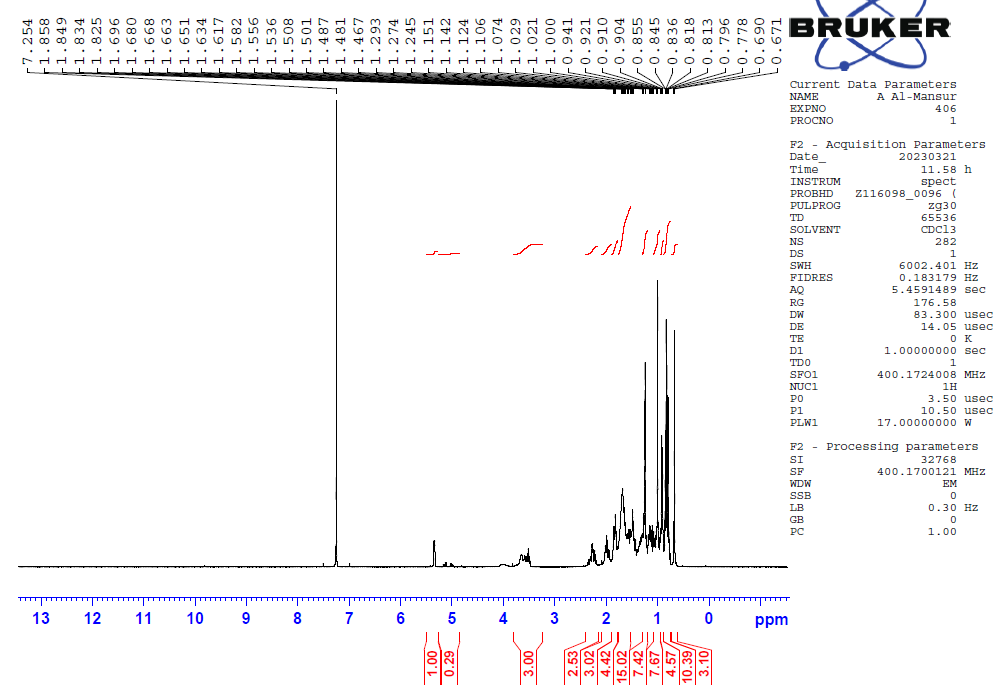


Figure S5: ^1^H NMR spectrum of compound **5** (β-sitosterol)
